# Serially Stacked Acellular Dermal Matrices in 2-Stage Implant-Based Breast Reconstruction

**DOI:** 10.1093/asjof/ojag024

**Published:** 2026-02-18

**Authors:** Jina Yom, Isabelle T Smith, Gabrielle P Odoom, Neil Tanna

## Abstract

Acellular dermal matrix (ADM) is widely used in implant-based breast reconstruction for implant stabilization, soft-tissue augmentation, and reduced capsular contracture, although its use in this setting remains off-label. Conventional approaches involve placing a single ADM layer during first-stage tissue expander placement. The authors present a novel approach that utilizes ADM in both stages of tissue expander/implant-based breast reconstruction, creating a bilayer ADM construct to enhance soft tissue support. This study aimed to describe the technique of serially stacked ADM in implant-based breast reconstruction and assess its preliminary safety profile. A retrospective review was conducted of all patients who received bilayer ADM in expander/implant-based breast reconstruction by a single surgeon between July 2020 and May 2024. Patient demographics, operative details, time between stages, follow-up duration, and major complications were assessed. Nineteen patients were included in this study. The mean age was 46 ± 8.6 years, and the mean BMI was 21.6 ± 2.85 kg/m^2^. Two patients had a history of radiation therapy. Seventeen reconstructions were prepectoral, and 2 were subpectoral. The average time from expander placement to implant exchange was 180 days. The average follow-up after the second stage was 10.8 months. No patients required reoperation or hospital readmission after either stage, and no major intraoperative or postoperative complications were reported. Preliminary findings from this cohort suggest that stacked ADM may be considered a safe and effective adjunct for enhancing soft tissue coverage in implant-based breast reconstruction. Larger prospective studies are needed to confirm these early results.

**Level of Evidence**: 4 (Therapeutic)

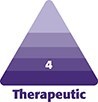

Prepectoral implant placement has emerged as a favorable alternative to the subpectoral approach in breast reconstruction because of reduced postoperative pain, shorter recovery, and avoidance of animation deformity. However, because the implant lies directly underneath the mastectomy skin envelope, prepectoral reconstruction is particularly susceptible to implant visibility, palpability, and rippling.^1,2^ Given the widespread use of acellular dermal matrix (ADM) to address these issues, serially stacked ADM may further enhance soft-tissue coverage in select patients.

The authors propose a novel technique of serially stacked ADM in 2-stage implant-based breast reconstruction. This technique utilizes an initial layer of ADM during tissue expander (TE) placement in the first stage of reconstruction, followed by wrapping the final implant with an additional ADM layer at the second stage to create a stacked ADM construct (Figure 1). The hypothesis of this innovation is that a bilayer ADM construct provides enhanced soft tissue coverage of the implant without significantly increasing the risk of complications, such as seroma, infection, or reconstruction failure.

**Figure 1.**
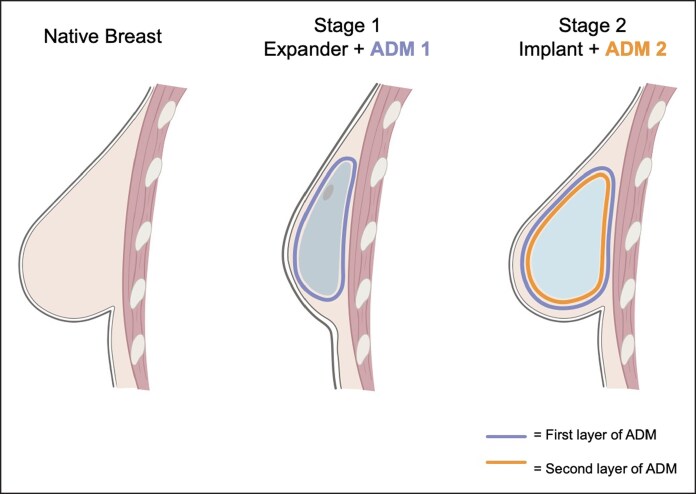
Illustration of the stages of serially stacked acellular dermal matrix (ADM) placement in implant-based breast reconstruction. The left panel illustrates the native breast. The middle panel shows Stage 1, in which the tissue expander is wrapped with the first layer of ADM (blue) and placed in the prepectoral plane. The right panel shows Stage 2, in which the tissue expander is removed and the final implant, wrapped in the second layer of ADM (orange), is placed in the prepectoral plane to create the bilayer ADM construct.

## METHODS

A retrospective chart review was conducted of all patients who received double-layered ADM in TE/implant-based breast reconstruction by a single surgeon (N.T.) between July 2020 and May 2024. This study was approved by the authors’ local IRB (IRB #: 23-0398). Patients with a history of radiation therapy, smoking, or BMI ≥40 were excluded. Patient demographics, operative details, and clinical outcomes were evaluated. The assessment of clinical outcomes was based primarily on clinical examination. Time between reconstruction stages and duration of follow-up were also measured. Complications were assessed, including rates of reoperation and hospital readmissions. Major complications were defined as any reoperation or rehospitalization within the first 90 days after either stage of surgery. Given the small sample size and preliminary nature of the study, statistical analysis focused primarily on mean, standard deviation, and range.

**Figure 2.**
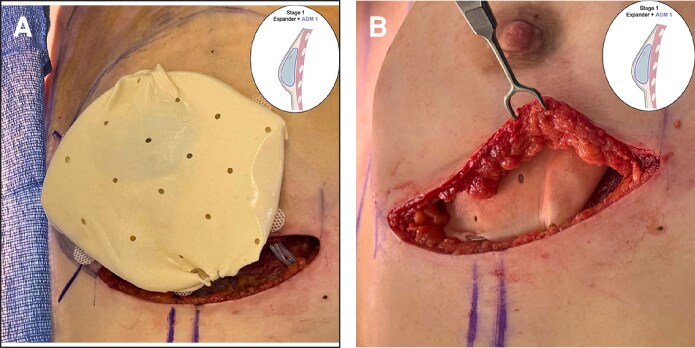
The first stage of the stacked acellular dermal matrix (ADM) technique is shown. The patient is a 27-year-old female. Following the left breast nipple-sparing mastectomy, the tissue expander is wrapped circumferentially in the first layer of ADM, (A) which is secured with interrupted sutures and (B) is placed into the prepectoral plane in the breast pocket (right).

**Figure 3.**
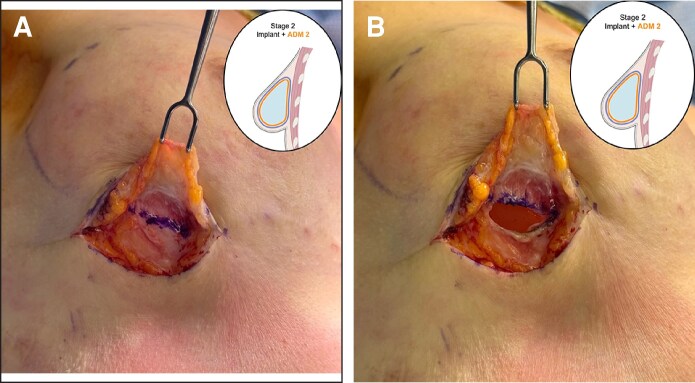
The second stage of the stacked acellular dermal matrix (ADM) technique is shown. The patient is a 27-year-old female. (A) At this stage, the previous mastectomy incision is reopened, revealing a well-vascularized capsule formed by the first ADM layer around the tissue expander. (B) An incision is then made in the capsule to enable removal of the tissue expander and placement of the final implant.

**Figure 4.**
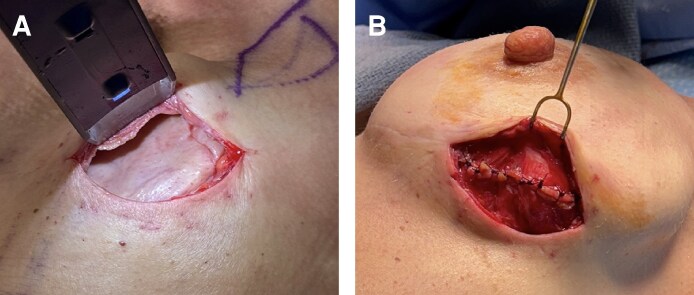
In another patient (a 38-year-old female), the second stage of the stacked acellular dermal matrix (ADM) technique is shown. (A) Following removal of the tissue expander, the ADM from Stage 1 is still present and appears to be integrated into the prepectoral breast pocket. (B) In Stage 2, after wrapping the final implant with the second layer of ADM, the implant-ADM construct is placed into the pocket within the capsule formed by the first layer of ADM (the patient is a 25-year-old female). The capsule is then sutured closed over the implant-ADM construct.

**Figure 5.**
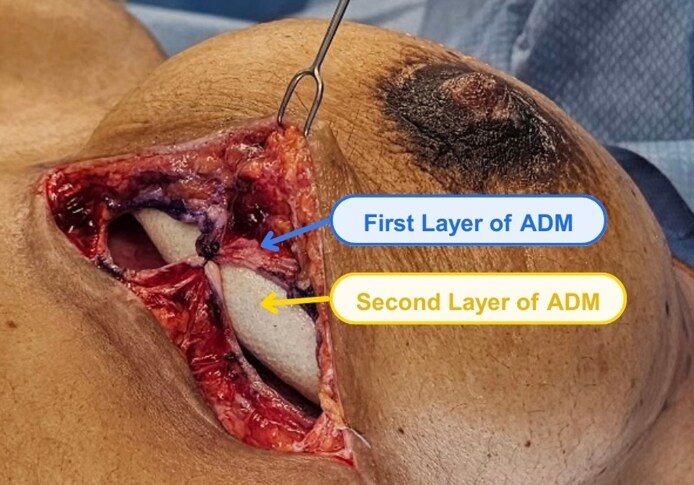
In this patient (a 41-year-old female), the bilayer acellular dermal matrix (ADM) construct at the time of final implant placement can be visualized. The well-vascularized first layer of ADM, placed at the time of initial-stage tissue expander placement (blue), is sutured to itself over the second layer of ADM that was placed with the final implant (orange).

For the stacked ADM construct, two 16 × 20 cm perforated rectangular sheets of ADM are utilized. The first-stage procedure entails a mastectomy followed by placement of a TE. After assessment of the mastectomy skin flaps through clinical assessment and intraoperative intravenous angiography and evaluation of the breast pocket, an appropriately sized TE and ADM of suitable thickness are selected. Then, the ADM is prepared, rinsed, circumferentially wrapped around the TE, and sutured to itself (Figure 2). The ADM-TE construct is secured to the chest wall in the prepectoral position (Figure 2). At the second surgical stage, the previously placed expander is removed with a capsulotomy (Figure 3). The final implant is selected based on breast dimensions and patient preference and is then wrapped with a second layer of ADM using the same technique as in the first stage. The implant-ADM construct is then placed in the prepectoral position, and the capsule from the first-stage procedure is repaired over the construct (Figures 4, 5). A closed suction drain is placed.

## RESULTS

A total of 19 female patients were included in this study. The mean age was 46 ± 8.6 years, the range of ages spanned from 27 to 63 years, and the mean BMI was 21.6 ± 2.85 kg/m^2^. Two patients received adjuvant postmastectomy radiation therapy. The average time between the first and second stages of reconstruction was 180 days, and the average follow-up time was 10.8 months. Most patients (*n* = 17) underwent prepectoral implant placement, whereas 2 patients had subpectoral implant placement because of a history of subpectoral breast augmentation. The implant sizes ranged from 220 to 520 cc, with an average volume of 357.7 cc. Almost all patients underwent bilateral nipple-sparing mastectomies (*n* = 18), with only one patient having a unilateral skin-sparing mastectomy. Eight patients underwent bilateral prophylactic mastectomies, whereas 11 patients underwent therapeutic mastectomies. No major intraoperative complications were reported. No major postoperative complications were reported, defined as any reoperation or hospital readmission within 90 days after either the first (TE placement) or second stage (implant placement) of surgery.

Three patients underwent a third procedure. One patient developed capsular contracture of the left breast 18 months after the second-stage surgery, requiring implant exchange without ADM. Notably, this patient received radiation therapy between the first and second stages, which may have impacted reconstruction outcomes. Another patient requested a smaller implant size for aesthetic reasons 3 years after second-stage reconstruction, resulting in implant exchange without ADM. Similarly, another patient requested a larger implant size for aesthetic reasons 18 months after the second stage, leading to implant exchange without ADM.

## DISCUSSION

Implant-based breast reconstruction has undergone much evolution, with continual development of new techniques aimed at improving aesthetic and patient-reported outcomes while minimizing complications. One longstanding point of debate in implant-based breast reconstruction is determining the optimal plane for implant placement. Prepectoral placement of the implant is challenged with issues of implant rippling, visibility, and palpability. Conversely, although subpectoral placement can mitigate some of these concerns, it has its own disadvantages, including animation deformity and increased postoperative pain because of muscle dissection.^1,2^ The advent of supportive materials such as ADM has facilitated prepectoral breast reconstruction by improving soft tissue coverage over the implant.

ADM has become a mainstay in implant-based breast reconstruction. Derived from processed and sterilized human, bovine, or porcine material, the allograft is designed to safely vascularize and incorporate into host native tissue.^3^ Although its use in this context is off-label, ADM is commonly used to optimize breast reconstruction and augmentation outcomes by reinforcing the breast pocket, stabilizing the implant position, reducing rates of capsular contracture, and correcting rippling.^4^ In TE reconstruction, ADM has also been shown to help prevent inferior “bottoming out” and lateral displacement.^5^ The allograft also allows for significantly higher initial TE fill volumes.^6^ The aesthetic benefits of ADM use in TE/implant breast reconstruction are well documented, with ADM-assisted reconstruction demonstrating higher scores in breast mound volume placement, inframammary fold definition, and aesthetic measures, such as contour, symmetry of size and shape, position, and overall appearance.^6-8^ However, some studies have described increased risks of complications such as infection and explantation associated with ADM use, although findings throughout the literature have been inconsistent.^5,6,8,9^

Historically, ADM has been utilized only at the first stage of implant-based reconstruction as a single-layer construct. Between the first and second stages of reconstruction, this ADM layer incorporates into the surrounding tissue of the breast pocket, creating a thick and well-vascularized soft tissue plane for the final implant placement. Second-stage reconstruction typically involves a simple TE-to-implant exchange. Histologic analyses at this stage have demonstrated ADM integration, with vascularization and the formation of a thin synovial metaplasia layer.^10^ Interestingly, when comparing single-layer ADM use to reconstruction without ADM, Marquez et al reported no differences in capsular contracture, implant rippling, or explantation rates.^11^

Recent efforts have explored innovative applications of ADM in breast reconstruction beyond traditional use in TE or direct-to-implant reconstruction. Gwak et al and Iskanderian et al both demonstrated the use of diced ADM pieces for volume replacement, specifically for filling defects after oncoplastic breast-conserving surgery.^12,13^ Lee et al evaluated the utility of a cylindrical rolled ADM construct to fill lumpectomy defects in oncoplastic breast-conserving surgery.^3^ Maxwell and Gabriel describe a technique using lower pole ADM in first-stage TE placement followed by upper pole, subpectoral placement of ADM at second-stage implant placement.^14^

**Figure 6.**
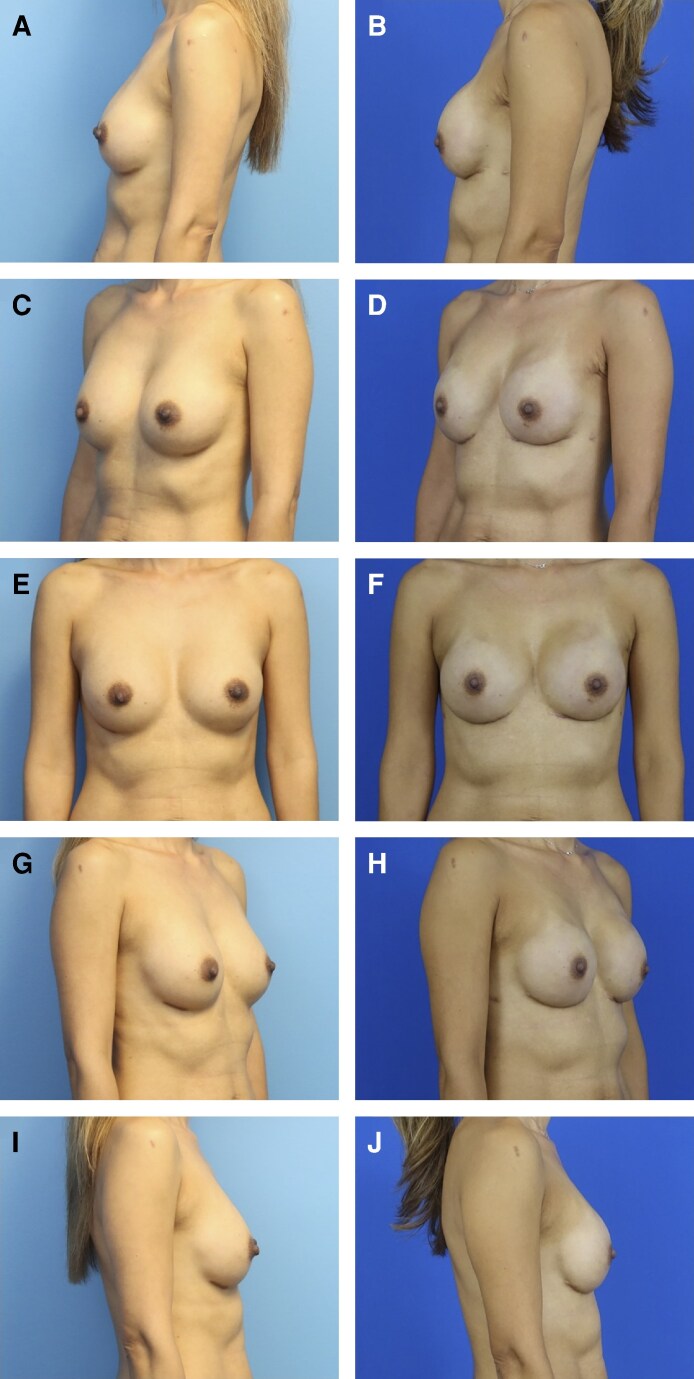
(A, C, E, G, I) Preoperative and (B, D, F, H, J) postoperative pictures in multiple views of a 49-year-old female patient following implant-based breast reconstruction with the stacked acellular dermal matrix technique.

The present study proposes a novel technique of serially stacked ADM placement, in which an additional layer of ADM is placed at second-stage reconstruction. By wrapping the final implant with a second ADM layer, this technique aims to further enhance soft tissue camouflage over the implant, potentially reducing implant visibility, palpability, and rippling. The use of additional ADM for volume replacement may also allow surgeons to utilize smaller implants, thereby decreasing final implant volume and surface area. Previous studies have reported that the use of thicker ADM increases risks of skin necrosis and infection, possibly because of delayed incorporation and neovascularization.^15^ The technique of serially stacked ADM aims to mitigate these concerns through the stepwise addition of ADM. Although seroma and infection remain known risks of ADM use, especially in cases of incomplete ADM incorporation into the surrounding tissue, the preliminary results from this cohort did not show an increased rate of such complications with use of a bilayer ADM construct. Based on anecdotal qualitative observations of differential ADM layer appearance at the 3 re-operations, the authors hypothesize that the outer layer of ADM may integrate, whereas the inner layer sits inert, similar to the underlying implant. However, this remains a hypothesis, because these observations are limited. Preoperative and postoperative comparisons in this cohort have shown excellent reconstructive outcomes (Figure 6).

Although the initial results from this novel technique are promising, there is much more to be explored. Limitations of this study include a small sample size and relatively short follow-up period. Although a control group with a single ADM wrap would enhance the paper, the authors have elected to not include the use of historical cases because of a temporal bias that would limit the validity of comparison. Future directions for studying this stacked ADM technique include formal evaluation of aesthetic and patient-reported outcomes, longer-term assessment of complications, and comparison with the traditional single-layer method. Additionally, future histologic and basic science studies are needed to evaluate the integration and vascularization patterns of each ADM layer within the surrounding pocket. Such analysis will be critical for understanding the implications of a thicker bilayer ADM construct on postoperative complications and long-term viability. Finally, although the use of stacked ADM introduces additional material costs, both layers were covered by insurance in this cohort. Given the nuanced cost and reimbursement landscape surrounding ADM use, a cost–benefit analysis for stacked ADM is warranted.

## CONCLUSIONS

The authors propose a novel technique of serially stacked ADM in 2-stage implant-based breast reconstruction. The technique utilizes an initial layer of ADM at TE placement during the first stage of reconstruction, followed by wrapping the final implant with an additional ADM layer at the second-stage procedure. In this initial retrospective review, no patients required reoperation or hospital readmission within 90 days after either stage of surgery, and no major intraoperative or postoperative complications were reported. These preliminary findings indicate that stacked ADM may be considered a safe and effective adjunct for enhancing soft tissue coverage in implant-based breast reconstruction. Larger prospective randomized studies are needed to formally confirm these early results.

## References

[1] Amro C, Sorenson TJ, Boyd CJ, et al The evolution of implant-based breast reconstruction: innovations, trends, and future directions. J Clin Med. 2024;13:7407. doi: 10.3390/jcm13237407 39685866 PMC11642416

[2] Frey J, Salibian A, Karp N, Choi M. Implant-based breast reconstruction: hot topics, controversies, and new directions. Plast Reconstr Surg. 2019;143:404e–416e. doi: 10.1097/PRS.0000000000005290 30688910

[3] Lee CB, Kim Y-S, Lee SE. Imaging features of volume replacement using an acellular dermal matrix in oncoplastic breast conserving surgery: a case report. Radiol Case Rep. 2022;17:2146–2149. doi: 10.1016/j.radcr.2022.03.003 35469303 PMC9034281

[4] Marra C, Cuomo R, Ceccaroni A, Pentangelo P, Alfano C. Acellular dermal matrix in breast augmentation surgery: a systematic review. JPRAS Open. 2024;40:111–117. doi: 10.1016/j.jpra.2024.02.004 38854623 PMC11156703

[5] Lanier ST, Wang ED, Chen JJ, et al The effect of acellular dermal matrix use on complication rates in tissue expander/implant breast reconstruction. Ann Plast Surg. 2010;64:674–678. doi: 10.1097/SAP.0b013e3181dba892 20395795

[6] Forsberg CG, Kelly DA, Wood BC, et al Aesthetic outcomes of acellular dermal matrix in tissue expander/implant-based breast reconstruction. Ann Plast Surg. 2014;72:S116–S120. doi: 10.1097/SAP.0000000000000098 24374398

[7] Nguyen KT, Mioton LM, Smetona JT, Seth AK, Kim JY. Esthetic outcomes of ADM-assisted expander-implant breast reconstruction. Eplasty. 2012;12:e58. 23308305 PMC3528352

[8] Ibrahim AMS, Koolen PGL, Ganor O, et al Does acellular dermal matrix really improve aesthetic outcome in tissue expander/implant-based breast reconstruction? Aesthetic Plast Surg. 2015;39:359–368. doi: 10.1007/s00266-015-0484-x 25894022

[9] Salibian AA, Bekisz JM, Kussie HC, et al Do we need support in prepectoral breast reconstruction? Comparing outcomes with and without ADM. Plast Reconstr Surg Glob Open. 2021;9:e3745. doi: 10.1097/GOX.0000000000003745 34386310 PMC8354628

[10] Bernini M, Gigliucci G, Cassetti D, et al Pre-pectoral breast reconstruction with tissue expander entirely covered by acellular dermal matrix: feasibility, safety and histological features resulting from the first 64 procedures. Gland Surg. 2024;13:297–306. doi: 10.21037/gs-23-432 38601291 PMC11002490

[11] Marquez JL, French M, Ormiston L, et al Outcomes after tissue expander exchange to implant in two-stage prepectoral breast reconstruction with and without acellular dermal matrix: a retrospective cohort study. J Plast Reconstr Aesthet Surg. 2024;89:97–104. doi: 10.1016/j.bjps.2023.12.008 38160591

[12] Gwak H, Jeon Y-W, Lim S-T, Park S-Y, Suh Y-J. Volume replacement with diced acellular dermal matrix in oncoplastic breast-conserving surgery: a prospective single-center experience. World J Surg Oncol. 2020;18:60. doi: 10.1186/s12957-020-01835-6 32209100 PMC7093974

[13] Iskanderian RR, Masri M, Nawaz N, Grobmyer SR. Acellular dermal matrix used for lumpectomy cavity volume replacement mimicking as breast cancer recurrence: a case report. Ann Breast Surg. 2021;5:20. doi: 10.21037/abs-20-93

[14] Maxwell GP, Gabriel A. Bioengineered breast: concept, technique, and preliminary results. Plast Reconstr Surg. 2016;137:415–421. doi: 10.1097/01.prs.0000475750.40838.53 26818275

[15] Nouri A, Nwaoz B, Ghosh K, et al Association between ADM thickness and complication risks in tissue expander breast reconstruction. Plast Reconstr Surg Glob Open. 2021;9:42–43. doi: 10.1097/01.GOX.0000799304.27835.5b

